# Pigment Dispersion Syndrome and Pigmentary Glaucoma: New Clinical Gradation and Current Therapeutic Strategies

**DOI:** 10.3390/jcm15020591

**Published:** 2026-01-12

**Authors:** Martyna Tomczyk-Socha, Artur Małyszczak, Anna Turno-Kręcicka

**Affiliations:** 1 Department of Ophthalmology, Wroclaw Medical University, Borowska 213, 50-556 Wrocław, Poland; 2 Department and Clinic of Ophthalmology, Wroclaw University Hospital, Borowska 213, 50-556 Wrocław, Poland

**Keywords:** pigment dispersion syndrome, pigmentary glaucoma, secondary glaucoma, clinical gradation

## Abstract

The classic triad of clinical findings in pigment dispersion syndrome was described decades ago. It consists of radial, spoke-like iris transillumination defects, pigment deposits on the corneal endothelium known as the Krukenberg spindle, and a densely and homogenously pigmented trabecular meshwork. PDS occurs approximately three times more frequently in young myopic men than in women and is most often identified between 30 and 50 years of age. The diagnostic evaluation does not differ from the standard examination performed in patients with suspected glaucomatous optic neuropathy. However, it must additionally incorporate examinations specific to PDS. The possible therapeutic approaches varying by disease stage will be discussed, including pharmacologic treatment, laser procedures (iridotomy and trabeculoplasty), and surgical approaches such as canaloplasty, trabeculectomy, and other glaucoma surgeries. In order to better identify patients requiring an optimal therapeutic strategy, we propose a division into five stages of PDS: (1) preclinical PDS, (2) visible PDS, (3) PDS converting to pigmentary glaucoma, (4) pigmentary glaucoma, and (5) inactive PDS. Therapeutic strategies of each stage are described below.

## 1. Introduction

Pigment dispersion syndrome (PDS) is characterized by the release of pigment from the posterior part of the iris, and its accumulation on the corneal endothelium and trabecular meshwork [1,2]. Overloaded trabecular meshwork limits aqueous humor outflow, which may result in increased intraocular pressure (IOP) and the development of pigmentary glaucoma (PG) [3]. It is estimated that 5 years after diagnosis of PDS, 10% of patients develop PG [4,5], but in the long term, it may affect up to one third of the PDS population [6]. That is why it is so important to continuously observe patients with PDS, monitor the occurrence of glaucomatous damage, and quickly initiate appropriate treatment. The aim of our work is to summarize current knowledge on the management of pigment dispersion syndrome and pigmentary glaucoma, and to propose a new clinical classification.

## 2. Pathophysiology and Clinical Characteristics

### 2.1. General Pathophysiology

Posterior bowing of the iris with “reverse pupillary block” configuration is noted in many eyes with PDS [5,7]. Iris concavity and a deep anterior chamber predispose to iridozonular contact, which causes pigment release during pupillary movement [1,8]. An additional factor promoting reverse pupillary block may be blinking, when aqueous fluid flows into the anterior chamber, pushing the iris backwards [9]. A combination of moderate myopia and increased anterior chamber depth provides a structural predisposition that promotes these pathophysiologic processes. Floating melanin granules or pigments may be seen in the anterior chamber as small brown molecules. The pigment accumulated in the anterior chamber gradually causes chronic injury, collapse, and alteration of the trabecular meshwork along with trabecular cells apoptosis. This impairs their function and efficiency. Other changes caused by pigment deposition at the filtration angle include fusion of trabecular lamellae and an increase in extracellular material, which, together with obliteration of the Schlemm canal, results in impaired outflow and increased IOP [10]. It has been shown that a higher amount of pigment particles in the anterior chamber is associated with higher IOP [11,12].

### 2.2. Genetic Background

PDS is suspected to have a genetic background. PDS has been described as an autosomal dominant condition with incomplete penetrance. A locus on chromosome 7q35–q36 has been implicated as a potential susceptibility region [13]. Genes that may be associated with PDS also include the premelanosome protein (PMEL), gamma secretase activator protein (GSAP), and glutamate metabotropic receptor 5 (GRM5) genes [14]. It is also suspected that myopia may have a direct causal effect on PDS, and that genes associated with lighter irises may also influence its occurrence [15,16]. The higher incidence of lattice degeneration may be related to congenital melanosome dysfunction [17].

### 2.3. PDS Clinical Characteristics

PDS is approximately three times more common in young myopic males than in females [18]. It is typically diagnosed between 30 and 50 years of age. PDS tends to stabilize with advancing age [19,20]. Risk factors include male gender, and moderate to severe myopia [5,21,22]. Myopia is a significant risk factor for PDS, and a higher degree of myopia may be associated with greater likelihood of transitioning to PG [21]. PDS can occur in normal phakic eyes, but also in pseudophakic eyes. The prevalence of PDS varies by race. It is four times higher in Caucasians than in Black or Asian populations, and the classic PDS phenotype in Caucasians is used as a benchmark [22]. The risk factors for PDS in the Black population are substantially different. In this group, PDS is associated with hyperopia, older age, convex iris, large lens, and a female predominance [23]. It is worth noting that iris transillumination, a typical sign of PDS in Caucasians, is rare in Black and Asian populations (the thick brown irides can make the detection of iris transillumination defects more difficult) [23,24].

In most cases, the patient does not experience any alarming symptoms. However, periodic pigment release may occur from pigmented epithelial cells located in the midperipheral iris [25]. Pigment release most often occurs in specific situations, such as physical exercise, being in a dark room with bright lights, e.g., watching a film at the cinema or attending a concert, frequent blinking, rapid head movements, or specific head positions. It may also occur during intense accommodation or stress [19,26]. Released pigment enters the anterior chamber during blinking and accumulates in the filtration angle [9,26]. This pigment causes blockage of the filtration angle and a marked increase in IOP, which may lead to eye pain, redness, photophobia, colored halos, and headaches. In addition, corneal oedema may occur, resulting in episodic, blurred vision. Large and frequent fluctuations in IOP can cause subsequent IOP elevations to become asymptomatic, despite substantial IOP increase. Therefore, patients may not experience eye pain or other disturbing symptoms when IOP rises, which means that PDS is often detected incidentally, for example when IOP is measured during spectacle prescription at an optician’s office or during an occupational health examination. These measurements often reach 50 mmHg or more.

## 3. New PDS/PG Clinical Gradation Scale

**Figure 1:**
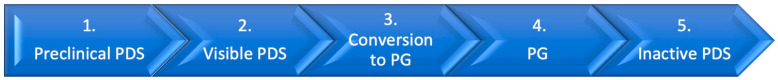
New clinical gradation scale of PDS.

The clinical course of PDS has traditionally been divided into three stages: pigment dispersion syndrome, conversion to PG, and pigment regression with possible normalization of IOP [27,28]. In order to better identify patients requiring an optimal therapeutic strategy, we propose a division into five stages (Figure 1):

1. Preclinical PDS—an initial stage of PDS with no clinical signs and symptoms. No IOP spikes are observed.
2. Visible PDS and glaucoma suspect—characterized by clinical signs and possible symptoms. IOP is usually within the normal range. IOP spikes may occur. No glaucomatous changes are present.
3. Converting PDS to pigmentary glaucoma—a transitional stage toward pigmentary glaucoma, with clinical signs and possible symptoms. IOP is elevated in most cases, and high-magnitude spikes may occur. No glaucomatous changes have yet developed.
4. Pigmentary glaucoma—secondary open-angle glaucoma.
5. Inactive PDS—with or without glaucoma. IOP control requires fewer medications. Less pigment release is observed. This stage can be misdiagnosed as Primary Open Angle Glaucoma (POAG).

### 3.1. Preclinical PDS

Preclinical PDS represents the initial pathophysiological stage at which PDS begins. Patients at this stage are otherwise healthy; however, a predisposition to pigment release can be detected. This phase may also be referred to as suspected PDS, although the term preclinical PDS more accurately reflects the underlying pathophysiology. The preclinical PDS is rarely identified in routine clinical practice.

In this stage of PDS, the patient does not have any signs and symptoms. IOP is in the normal range, with no IOP spikes. The pigment begins to accumulate but does not yet meet the characteristic features of PDS (on gonioscopy, the trabecular meshwork is more saturated with pigment but is not yet homogenously intensely dark, and there is no Krukenberg spindle or iris transillumination). The pigment release is limited, so the free pigment is not visible in the anterior chamber. Posterior iris bowing can be observed in gonioscopy and AS-OCT, as a condition predisposing to further pigment release.

### 3.2. Visible PDS and Glaucoma Suspect

The classic triad of clinical signs was identified over 75 years ago in visible PDS. To this day, it remains the basis for the diagnosis of classic PDS [29,30]. It includes 1. spoke-like iris transillumination, 2. pigment clusters on the corneal endothelium, called Krukenberg spindles, and 3. a heavily homogenously pigmented trabecular meshwork [30].

Midperipheral iris transillumination with a radial, spoke-like pattern due to pigment loss is best visible with retroillumination. Peripheral transillumination reflects loss of pigment on the posterior surface of the iris. This loss is caused by contact and rubbing of the iris against the zonular fibers, followed by pigment release. These defects are not pathognomonic for PDS but are observed in most cases, although they may by absent in dark-pigmented irides.

A frequently seen Krukenberg spindle is a vertically oriented accumulation of pigment deposits on the endothelium. The shape of the spindle is generated by the movement of aqueous humor in the anterior chamber, convection currents, and subsequent pigment phagocytosis by endothelial cells. Histologic analyses indicate that melanin granules are internalized by endothelial cells through phagocytosis rather than merely accumulating as superficial deposits [30]. The Krukenberg spindle is not pathognomic for PDS. It is seen also in pseudoexfoliation syndrome and other types of glaucoma.

**Figure 2:**
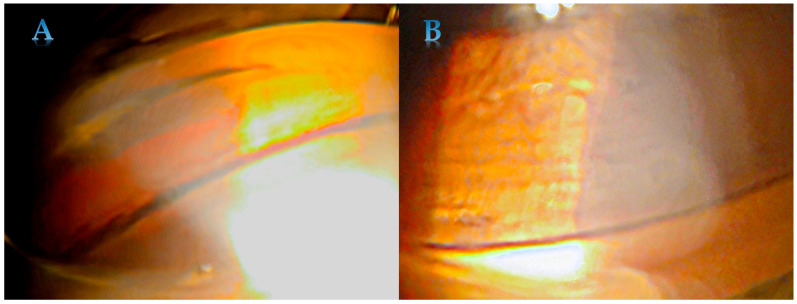
Homogenous and intense pigmentation of trabecular meshwork in PDS—in the lower (**A**) and upper part (**B**).

Pigmentation of the trabecular meshwork is typically homogenous and intense (Figure 2). The pigmentation is more advanced in the lower part of the drainage angle. Pigment can create a deposition near Schwalbe’s line called the Sampaolesi line. The Sampaolesi line is associated primarily with pseudoexfoliation syndrome [31], but it may also be seen in PDS. Pigment deposition along the insertion of the zonular fibers into the lens is described as a ‘Scheie stripe’ or ‘Zentmayer’s ring’. While corneal endothelial polymegathism and pleomorphism have been observed in PDS, there is no evidence that central corneal thickness or endothelial pump function are significantly affected [32].

Not all symptoms from the classic triad need to be present in patients with PDS. In the Caucasian population, Krukenberg spindle was described in 95% of patients, trabecular meshwork pigmentation in 86%, and iris transillumination defects in 86% [4]. In the most recent research on Caucasian patients, the prevalence was comparable for Krukenberg spindle and trabecular meshwork pigmentation (91%, 88.9%), but iris transillumination defects were much less common, with a prevalence of 24.3% [33]. In the other study, transillumination defects were present in 95% [34]. Anisocoria may occur in some cases, reflecting asymmetric mydriasis caused by mechanical irritation of the iris smooth muscle in areas of iris–lens contact [35]. A characteristic feature is also a deep anterior chamber. Lattice degeneration was found in one third of eyes with PDS [36]. Lattice degeneration may be not directly related to PDS, but rather to myopia which is common among patients with PDS.

### 3.3. Conversion to Pigmentary Glaucoma

High risk of progression to PG occurs when the trabecular meshwork is heavily filled with pigment. The resistance of aqueous outflow increases, and this causes a sustained increase in IOP. Elevated IOP can lead to glaucomatous changes. Other factors associated with an increased likelihood of conversion from PDS to PG include family history, myopia, Caucasian population, male sex, a Krukenberg spindle, increased initial IOP, and a PDS diagnosis persisting for more than five years [37]. PDS accounts for 1–1.5% of all glaucoma cases [5]. Siddiqui described the risk of conversion to pigmentary glaucoma as 10% at 5 years and 15% in 15 years [4].

### 3.4. Pigmentary Glaucoma

The presence of glaucomatous damage results in a diagnosis of secondary glaucoma due to PDS—pigmentary glaucoma. Compared to POAG, PG occurs at a much younger age [2]. Although the changes in the optic nerve do not differ from those caused by POAG, PG has a different etiology, which significantly influences the diagnostic and therapeutic approach. The different etiology also affects the prognosis, which appears to be more favorable than in POAG, with total blindness rarely occurring as a result of PG [6,38]. Nevertheless, this does not diminish the risk associated with the disease, which primarily affects a younger population, with visual field defects being equally irreversible.

Some patients already have optic nerve neuropathy at the time of PDS diagnosis. The treatment strategy in such cases follows classical principles—if there is glaucomatous damage and elevated IOP, intraocular pressure should be lowered.

### 3.5. Inactive PDS

PDS or PG may become milder over time. The older the patient, the lower the IOP and the slower the progression of the disease is observed. Migliazzo et al. found, in their 17-year observation, that in 28% of patients, glaucoma control required fewer medications in later years [6]. There are several hypotheses explaining this phenomenon. With age, increasing lenticular thickness may pull the iris away from the zonules, reducing the risk of mechanical contact. It is also possible that the pigment does not regenerate, so further contact does not cause additional release. Drainage angle is less heavily pigment inferiorly compared to superiorly (pigment reversal sign) and there are no IOP spikes [39]. All this results in less pigment being released from the iris, which ultimately leads to a reduction in IOP. Age-related declines in aqueous humor production may also have an impact [19].

## 4. Diagnostic Process and Examination

The aim of the diagnostic process is to confirm PDS and determine whether glaucomatous damage to the optic nerve has occurred, and whether PG has developed. The diagnostic process does not differ from the standard examination of a patient suspected of having glaucomatous neuropathy, but it should also include tests specific to PDS. When performing gonioscopy, attention should be paid to the amount of pigment in the filtration angle and the configuration of the iris, including whether reverse pupillary block is possible. During the examination of the anterior segment, a retroillumination examination should be performed to identify characteristic pigment defects on the iris, and the endothelium should be checked for pigment arranged in a vertical line. During pupillary dilation, the lens and ciliary fibers should be assessed for the presence of pigment. Anterior segment optical coherence tomography (AS-OCT) typically demonstrates an enlarged irido-lenticular contact area, increased anterior chamber volume and depth, as well as an enlarged trabecular-iris space area (TISA) [39]. Ultrasound biomicroscopy and AS-OCT provide reliable imaging for the morphological assessment of posterior iris bowing [40,41]. The phenylephrine provocative test is used to screen for susceptibility to IOP spikes. A 10% phenylephrine drop is administered three times at five-minute intervals, followed by assessment of pigment dispersion into the anterior chamber. IOP measurements are performed at the first and second hour post-instillation to monitor for pressure elevation and to initiate treatment if needed [42].

## 5. Therapeutic Strategies

Although there is no specific treatment for PDS or PG, the effectiveness of available methods may vary across different PDS/PG stages. The main approaches include pharmacological treatment, laser peripheral iridotomy (LPI) using YAG laser, laser trabeculoplasty: argon laser trabeculoplasty (ALT), selective laser trabeculoplasty (SLT), micropulse laser trabeculoplasty (MLT), and surgical treatment. The potential therapeutic strategies appropriate for each stage of the disease will be discussed below.

### 5.1. Pharmacological Treatment

Traditionally, PDS or PG has been treated with miotics, primarily pilocarpine [2,19]. Pilocarpine is theoretically an option for patients with PG. It reduces pupil movement (miosis) and decreases posterior iris bowing, but due to its side effects, including headache, accommodative spasms, blurred vision, and increased risk of retinal detachment, it is rarely used in current practice.

Pharmacological treatment now includes standard substances used to lower IOP. Currently, these comprise substances from five groups: beta blockers, alpha 2 mimetics, carbonic anhydrase inhibitors, prostaglandin analogues, and RHO kinase inhibitors. Among pharmacological treatments, prostaglandin analogues are traditionally the first choice, as they increase the outflow of aqueous humor through an unconventional route, where it passes through the ciliary muscle and into the supraciliary space. A randomized clinical trial reported that once-daily latanoprost provided more effective and better-tolerated one-year IOP control in PG than twice-daily timolol [43]. There is a growing hypothesis that the non-conventional outflow of aqueous humor also involves lymphatic pathways and that prostaglandin analogues may support this uveolymphatic pathway [44,45]. Selective α2-agonists may be useful in PG, given their potential increased hypersensitivity of adrenergic receptors in these patients [3].

### 5.2. Laser Treatment

#### 5.2.1. Laser Peripheral Iridotomy (LPI)

One of the bases of PG pathophysiology is reverse pupillary block and an imbalance in aqueous humor pressure between the anterior and posterior chambers. This results in iridocyclic contact and facilitates pigment release. LPI creates an additional pathway between the chambers, equalizing pressure and reducing iris concavity [46,47,48]. Indeed, it has been shown that LPI performed in patients with PDS reduces the amount of aqueous melanin granules [49]. Unfortunately, despite promising morphological results, there is limited evidence for the long-term effectiveness of this procedure in inhibiting the progression of visual field defects. Labbé et al. conducted a literature review on the effectiveness of LPI in PDS and PG [50]. The review included eight trials with a total of 218 participants. LPI resulted in reduced IOP in PG patients, but had no effect on the risk of progression after a mean of 28 months. None of the analyzed studies demonstrated the effectiveness of LPI in reducing the risk of glaucoma conversion in individuals with PDS [50]. In a Cochrane review published in 2016, the authors concluded that none of the five included RCTs showed clear efficacy of LPI in preventing the progression of visual field defects in patients with PG or PDS [51]. They also noted discrepancies in reporting across studies, which prevented them from performing a meta-analysis [51]. Perhaps the key to the effectiveness of LPI lies in the appropriate selection of patients for the procedure. Gandolfi et al. used a phenylephrine test to identify, among patients with PDS, those with a high risk of IOP decompensation [52]. They performed LPI in randomly selected eyes of these patients. During a 10-year follow-up, it resulted in a reduction in the risk of IOP increase to a level comparable to that observed in the low-risk group. It is worth mentioning that the average age of the subjects in each group was below 35 years [52]. In another study, LPI was performed in patients with PDS and, in a 2-year follow-up, resulted in a lower risk of IOP increase. This effect was mainly observed in subjects younger than 40 years of age [53]. The reduction in iridociliary contact caused by LPI is probably most important during the active phase of pigment release. Unfortunately, despite the potential patophysiological basis for the effectiveness of LPI, and reports of effective IOP reduction in some patients, the available studies do not show the effectiveness of this therapy in inhibiting PG progression. Therefore, LPI may be an option in individuals under 40 years, with evident iridozonular contact and normal IOP without glaucoma changes.

#### 5.2.2. Laser Trabeculoplasty

Laser trabeculoplasty stimulates and alters the function of trabecular meshwork cells, which results in increased aqueous outflow. It is an effective treatment for POAG, in which it may even be considered a first line therapy [5]. Both ALT and SLT are effectively used to treat PG. ALT is an older procedure, and more data on its effectiveness is available. Reports show varying lengths of ALT effect duration, but typically not exceeding three years [54,55]. Importantly, the effect of treatment appears to be longer in younger patients [54,56]. Ritch at al. reported ALT success rates of 45% after six years [54]. The benefit of SLT over ALT is its better safety profile, while its effect duration is similar. In a retrospective analysis of PG patients undergoing SLT, the therapeutic effect lasted up to 2 years. SLT is a repeatable procedure, which is a significant advantage considering the continuous release of pigment and its accumulation in the iridocorneal angle. Ayala at al. estimated the average time to failure after SLT at around 27 months [57].

However, it should be remembered that a highly pigmented TM in PDS may cause IOP spikes. Other risk factors include multiple glaucoma medications and a history of ALT. Hallaj et al. reported that SLT was comparably effective in PG and POAG, but IOP spikes were observed only in the PG group, despite the lower laser energy used [58]. Therefore, it is suggested to reduce the laser dose during treatment and to use prophylactic treatment to prevent IOP spikes after laser treatment (drops in lower pre-treatment IOP or oral acetazolamide in high-risk groups and higher pre-treatment IOP) [57,59,60]. The newest trabeculoplasty technique, MLT, is also less traumatic than ALT and has demonstrated efficacy comparable to SLT in controlling IOP. Studies involving large groups of patients confirm that MLT is as effective as SLT, but they did not include adequate numbers of PG patients [61].

### 5.3. Surgical Treatment

Surgery is also successfully used in the treatment of PDS. Given the young age of patients with PDS and the long-expected duration of glaucoma, earlier consideration for surgical intervention is often warranted.

Trabeculectomy has been shown to be an effective treatment method. In an 8-year follow-up of patients with a mean baseline age of 35.5 years, trabeculectomy stabilized IOP and halted the progression of visual field defects [62]. PDS is a favorable prognostic factor in the postoperative course; however, there are reports of an increased risk of bleb infections [63,64]. Young myopic males also have a higher risk of hypotony, maculopathy, and suprachoroidal hemorrhage after any bleb-forming surgery. Other methods of glaucoma surgery also appear to be effective. Akil et al. described similar outcomes of ab interno trabeculectomy using the Trabectome in patients with POAG and PG [65]. The effectiveness of XEN45 gel stent implantation and canaloplasty has also been demonstrated [66,67,68]. It is worth noting that in both aforementioned studies, pigmentation loss was observed after the procedures [62,68]. Brusini et al. suggested that the accelerated elimination of pigment, before trabeculum is significantly impaired, may be the reason for the long-term effectiveness of the therapy [68]. The efficacy of trabecular micro-bypass stent implantation in pigmentary glaucoma remains uncertain. Klammann et al. reported poorer postoperative IOP control in PG compared with POAG and pseudoexfoliation glaucoma [69]. Conversely, in a retrospective series, Ferguson et al. found that iStent implantation combined with cataract extraction maintained IOP below 18 mmHg at three years in 95% of PG eyes [70]. Jacobi et al. found that trabecular aspiration provided minimal benefit in PG, with a one-month success rate of just 12% [71]. Cataract surgery in older patients eliminates lens iris rubbing as well, so early removal of the cataractous lens is recommended [72].

## 6. Treatment and Management at Every Stage of PDS

- Stage 1—Preclinical PDS

No treatment required, routine observation is recommended.

- Stage 2—Visible PDS and glaucoma suspect

This is a group of patients who do not yet have glaucomatous neuropathy and for whom every decision is accompanied by doubts and risks. If IOP is normal and we only observe patients, how can we be sure that IOP does not increase significantly during physical exertion or when they are in a darkened room? Individuals with PDS at increased risk of conversion to PG are recommended to have more frequent periodic assessments of IOP and optic nerve head morphology. In younger patients with visible reverse pupillary block, LPI may be considered.

- Stage 3—Converting PDS to pigmentary glaucoma

Patients in group three should be monitored particularly closely, as morphological changes in the filtration angle and progressively increasing IOP may lead to glaucomatous changes and conversion to PG. Such patients should be monitored every three to six months. Performing a visual field test is often easier for these young patients and requires less effort than for typically older glaucoma patients. Therefore, it should be performed regularly and frequently. IOP measurements are often overestimated with non-contact tonometry methods, so Goldman applanation tonometry (GAT) should be used. IOP measurements should be taken frequently and at different times of the day. Each 1 mmHg increase in IOP may increase the risk of developing PG by 1.4 times [4]. In stage 3, LPI may be considered to reduce the amount of pigment released. If there are documented periodic increases in IOP or if IOP is substantially higher than 21 mmHg SLT or the initiation of pharmacological treatment should be considered. If IOP decompensation occurs with values significantly above normal, surgical treatment should also be considered.

- Stage 4—Pigmentary Glaucoma

As in other forms of open-angle glaucoma, therapy is usually initiated when high IOP coincides with demonstrable structural and/or functional glaucomatous changes. In some patients with PDS, IOP may not be elevated during the visit, but frequent IOP spikes can damage the optic nerve. Therefore, if glaucoma progression is evident, treatment to lower IOP should be initiated even if IOP is normal during visits. The management of PG can be similar to that of other glaucoma types. As described in Section 5.1, prostaglandin analogues are traditionally the first choice. In addition to local IOP-lowering therapy, patients often require surgical treatment. When deciding on the best treatment method for a given patient, life expectancy and quality of life should be taken into account. As the diagnosis is most common in young adults who will require long-term management, surgical treatment may be initiated quicker than in POAG.

- Stage 5—Inactive PDS

Only observation is required, and treatment may be reduced, when possible.

Treatment depended on stage as summarized in Table 1.

## 7. Conclusions

We presented a new clinical gradation system of PDS and discussed treatment strategies in each stage. Due to the various stages of Pigment Dispersion Syndrome, there is no universal treatment method. It is necessary to find a “middle ground” in the treatment of each patient, considering all possible methods, and taking into consideration the stage of the disease. In advanced disease, aggressive surgical treatment may be a better solution, while in the early stages, observation or conservative treatment is often more appropriate. In addition, laser procedures such as ALT, SLT, and MLT appear to be reasonable, effective and, for the last two, repeatable treatment options for PDS. Qualification for LPI should be approached with caution, as its effectiveness in preventing conversion to PG has not been proven. Due to the multiple possible treatment methods, in some cases, we may only be able to draw conclusions about the correctness of our approach retrospectively. Therefore, more research is needed to determine the most appropriate management strategies in PDS.

## Figures and Tables

**Table 1:** Stage-depended PDS/PG treatment.

Stage	Characteristics	Suggested Management
1—Preclinical PDS	No clinical signs or symptoms	None
2—Visible PDS and glaucoma suspect	Clinical signs are present IOP usually within the norm, spikes may occur	More frequent examinations in patients with risk factors * LPI may be considered in younger populations with visible reverse pupillary block
3—PG conversion	Clinical signs are present Patient may be symptomatic IOP elevated in most cases, high magnitude spikes may occur	Examination every 3–6 months If IOP is substantially higher than 21 mmHg, consider trabeculoplasty or topical treatment or, in severe cases, surgical treatment
4—Pigmentary Glaucoma	Glaucomatous changes are present	Similar to POAG Among pharmacological treatments, consider prostaglandin analogs If SLT is considered, use lower laser energy Surgical treatment may be initiated quicker than in POAG
5—Inactive PDS/PG	IOP control requires fewer medications No further pigment release	Reduction in treatment may be considered

* Risk factors include IOP spikes, family history, myopia, Caucasian population, male sex, Krukenberg spindle, and PDS diagnosis for more than 5 years.

## Data Availability

No new data were created or analyzed in this study. Data sharing is not applicable.

## Abbreviations

The following abbreviations are used in this manuscript:

PDS	Pigment Dispersion Syndrome
IOP	IntraOcular Pressure
PG	Pigmentary Glaucoma
POAG	Primary Open Angle Glaucoma
AS-OCT	Anterior Segment Optical Coherence Tomography
LPI	Laser Peripheral Iridotomy
ALT	Argon Laser Trabeculoplasty
SLT	Selective Laser Trabeculoplasty
MLT	Micropulse Laser Trabeculoplasty

## References

[1] Campbell D.G., Schertzer R.M. (1995). Pathophysiology of Pigment Dispersion Syndrome and Pigmentary Glaucoma: Editorial Review. Curr. Opin. Ophthalmol..

[2] Farrar S.M., Shields M.B. (1993). Current Concepts in Pigmentary Glaucoma. Surv. Ophthalmol..

[3] Okafor K., Vinod K., Gedde S.J. (2017). Update on Pigment Dispersion Syndrome and Pigmentary Glaucoma. Curr. Opin. Ophthalmol..

[4] Siddiqui Y., Ten Hulzen R.D., Cameron J.D., Hodge D.O., Johnson D.H. (2003). What Is the Risk of Developing Pigmentary Glaucoma from Pigment Dispersion Syndrome?. Am. J. Ophthalmol..

[5] Pazos M., Traverso C.E., Viswanathan A., European Glaucoma Society, The Guidelines Task Force, The Guidelines Writers, Authors and Contributors, The Guidelines Internal Reviewers, The Experts by Experience Group (Patients’ Panel), Team of Clinica Oculistica of the University of Genoa for Medical Editing and Graphics, External Reviews (2025). European Glaucoma Society—Terminology and Guidelines for Glaucoma, 6th Edition. Br. J. Ophthalmol..

[6] Migliazzo C.V., Shaffer R.N., Nykin R., Magee S. (1986). Long-Term Analysis of Pigmentary Dispersion Syndrome and Piegmentary Glaucoma. Ophthalmology.

[7] Sokol J., Stegman Z., Liebmann J.M., Ritch R. (1996). Location of the Iris Insertion in Pigment Dispersion Syndrome. Ophthalmology.

[8] Davidson J.A., Brubaker R.F., Ilstrup D.M. (1983). Dimensions of the Anterior Chamber in Pigment Dispersion Syndrome. Arch. Ophthalmol..

[9] Liebmann J.M., Tello C., Chew S.-J., Cohen H., Ritch R. (1995). Prevention of Blinking Alters Iris Configuration in Pigment Dispersion Syndrome and in Normal Eyes. Ophthalmology.

[10] Gottanka J., Johnson D.H., Grehn F., Ltjen-Drecoll E. (2006). Histologic Findings in Pigment Dispersion Syndrome and Pigmentary Glaucoma. J. Glaucoma.

[11] Mardin C.Y., Küchle M., Nguyen N.X., Martus P., Naumann G.O.H. (2000). Quantification of Aqueous Melanin Granules, Intraocular Pressure and Glaucomatous Damage in Primary Pigment Dispersion Syndrome. Ophthalmology.

[12] Alvarado J.A. (1992). Outflow Obstruction in Pigmentary and Primary Open Angle Glaucoma. Arch. Ophthalmol..

[13] Andersen J.S. (1997). A Gene Responsible for the Pigment Dispersion Syndrome Maps to Chromosome 7q35-q36. Arch. Ophthalmol..

[14] Rong S., Yu X., Wiggs J.L. (2024). Genetic Basis of Pigment Dispersion Syndrome and Pigmentary Glaucoma: An Update and Functional Insights. Genes.

[15] Simcoe M.J., Shah A., Fan B., Choquet H., Weisschuh N., Waseem N.H., Jiang C., Melles R.B., Ritch R., Mahroo O.A. (2022). Genome-Wide Association Study Identifies Two Common Loci Associated with Pigment Dispersion Syndrome/Pigmentary Glaucoma and Implicates Myopia in Its Development. Ophthalmology.

[16] Simcoe M.J., Weisschuh N., Wissinger B., Hysi P.G., Hammond C.J. (2020). Genetic Heritability of Pigmentary Glaucoma and Associations With Other Eye Phenotypes. JAMA Ophthalmol..

[17] Scuderi G., Papale A., Nucci C., Cerulli L. (1996). Retinal Involvement in Pigment Dispersion Syndrome. Int. Ophthalmol..

[18] Di Pippo M., Ciancimino C., Scuderi L., Perdicchi A. (2020). An Iconic Case of Pigmentary Glaucoma: Brief Review of the Literature. Case Rep. Ophthalmol..

[19] Niyadurupola N., Broadway D.C. (2008). Pigment Dispersion Syndrome and Pigmentary Glaucoma—A Major Review. Clin. Exper Ophthalmol..

[20] Orgül S., Hendrickson P., Flammer J. (1994). Anterior Chamber Depth and Pigment Dispersion Syndrome. Am. J. Ophthalmol..

[21] Farrar S.M., Shields M.B., Miller K.N., Stoup C.M. (1989). Risk Factors for the Development and Severity of Glaucoma in the Pigment Dispersion Syndrome. Am. J. Ophthalmol..

[22] Pang R., Labisi S.A., Wang N. (2023). Pigment Dispersion Syndrome and Pigmentary Glaucoma: Overview and Racial Disparities. Graefe’s Arch. Clin. Exp. Ophthalmol..

[23] Semple H.C., Ball S.F. (1990). Pigmentary Glaucoma in the Black Population. Am. J. Ophthalmol..

[24] Qing G., Wang N., Tang X., Zhang S., Chen H. (2009). Clinical Characteristics of Pigment Dispersion Syndrome in Chinese Patients. Eye.

[25] Kupfer C., Kuwabara T., Kaiser-Kupfer M. (1975). The Histopathology of Pigmentary Dispersion Syndrome with Glaucoma. Am. J. Ophthalmol..

[26] Karickhoff J.R. (1992). Pigmentary Dispersion Syndrome and Pigmentary Glaucoma: A New Mechanism Concept, a New Treatment, and a New Technique. Ophthalmic Surg..

[27] Yamashita T., Shiihara H., Terasaki H., Fujiwara K., Tanaka M., Sakamoto T. (2022). Characteristics of Pigmentary Glaucoma in Japanese Individuals. PLoS ONE.

[28] Speakman J.S. (1981). Pigmentary Dispersion. Br. J. Ophthalmol..

[29] Sugar H.S., Barbour F.A. (1949). Pigmentary Glaucoma; a Rare Clinical Entity. Am. J. Ophthalmol..

[30] Saul Sugar H. (1966). Pigmentary Glaucoma. A 25-Year Review. Am. J. Ophthalmol..

[31] Iwanejko M., Turno-Kręcicka A., Tomczyk-Socha M., Kaczorowski K., Grzybowski A., Misiuk-Hojło M. (2017). Evaluation of the Anterior Chamber Angle in Pseudoexfoliation Syndrome. Adv. Clin. Exp. Med..

[32] Lehto I., Ruusuvaara P., Setälä K. (1990). Corneal Endothelium in Pigmentary Glaucoma and Pigment Dispersion Syndrome. Acta Ophthalmol..

[33] Benitez-del-Castillo J., Villalba-Conde M., Amaya-López V., Pinazo-Duran M.D. (2025). Pigmentary Dispersion Syndrome and Pigmentary Glaucoma: Diagnostic Relevance of the Classical Triad in a Mediterranean Population. Arch. Soc. Española Oftalmol. (Engl. Ed.).

[34] Doane J.F., Rickstrew J.J., Tuckfield J.Q., Cauble J.E. (2019). Prevalence of Pigment Dispersion Syndrome in Patients Seeking Refractive Surgery. J. Glaucoma.

[35] Feibel R.M., Perlmutter J.C. (1990). Anisocoria in the Pigmentary Dispersion Syndrome. Am. J. Ophthalmol..

[36] Lehto I., Vesti E. (1998). Diagnosis and Management of Pigmentary Glaucoma. Curr. Opin. Ophthalmol..

[37] Zeppieri M. (2022). Pigment Dispersion Syndrome: A Brief Overview. J. Clin. Transl. Res..

[38] Lehto I. (1991). Long-term Prognosis of Pigmentary Glaucoma. Acta Ophthalmol..

[39] Bustamante-Arias A., Ruiz-Lozano R.E., Carlos Alvarez-Guzman J., Gonzalez-Godinez S., Rodriguez-Garcia A. (2021). Pigment Dispersion Syndrome and Its Implications for Glaucoma. Surv. Ophthalmol..

[40] Potash S.D., Tello C., Liebmann J., Ritch R. (1994). Ultrasound Biomicroscopy in Pigment Dispersion Syndrome. Ophthalmology.

[41] Birner B., Tourtas T., Wessel J.M., Jünemann A.G., Mardin C.Y., Kruse F.E., Laemmer R. (2014). Melanindispersionssyndrom und -glaukom: Morphometrische Analyse des vorderen Augenabschnittes mittels SL-OCT. Ophthalmologe.

[42] Epstein D.L., Boger W.P., Morton Grant W. (1978). Phenylephrine Provocative Testing In The Pigmentary Dispersion Syndrome. Am. J. Ophthalmol..

[43] Mastropasqua L., Carpineto P., Ciancaglini M., Gallenga P.E. (1999). A 12-Month, Randomized, Double-Masked Study Comparing Latanoprost with Timolol in Pigmentary Glaucoma. Ophthalmology.

[44] Tomczyk-Socha M., Turno-Kręcicka A. (2017). A Novel Uveolymphatic Drainage Pathway—Possible New Target for Glaucoma Treatment. Lymphat. Res. Biol..

[45] Kim Y.K., Na K.I., Jeoung J.W., Park K.H. (2017). Intraocular Pressure-Lowering Effect of Latanoprost Is Hampered by Defective Cervical Lymphatic Drainage. PLoS ONE.

[46] Carassa R.G., Bettin P., Fiori M., Brancato R. (1998). Nd:YAG Laser Iridotomy in Pigment Dispersion Syndrome: An Ultrasound Biomicroscopic Study. Br. J. Ophthalmol..

[47] Laemmer R., Mardin C.Y., Juenemann A.G.M. (2008). Visualization of Changes of the Iris Configuration After Peripheral Laser Iridotomy in Primary Melanin Dispersion Syndrome Using Optical Coherence Tomography. J. Glaucoma.

[48] Breingan P.J. (1999). Iridolenticular Contact Decreases Following Laser Iridotomy for Pigment Dispersion Syndrome. Arch. Ophthalmol..

[49] Küchle M., Nguyen N.X., Mardin C.Y., Naumann G.O. (2001). Effect of Neodymium:YAG Laser Iridotomy on Number of Aqueous Melanin Granules in Primary Pigment Dispersion Syndrome. Graefe’s Arch. Clin. Exp. Ophthalmol..

[50] Buffault J., Leray B., Bouillot A., Baudouin C., Labbé A. (2017). Role of Laser Peripheral Iridotomy in Pigmentary Glaucoma and Pigment Dispersion Syndrome: A Review of the Literature. J. Français d’Ophtalmol..

[51] Michelessi M., Lindsley K.B. (2016). Peripheral Iridotomy for Pigmentary Glaucoma. Cochrane Database Syst. Rev..

[52] Gandolfi S.A., Ungaro N., Tardini M.G., Ghirardini S., Carta A., Mora P. (2014). A 10-Year Follow-up to Determine the Effect of YAG Laser Iridotomy on the Natural History of Pigment Dispersion Syndrome: A Randomized Clinical Trial. JAMA Ophthalmol..

[53] Gandolfi S.A., Vecchi M. (1996). Effect of a YAG Laser Iridotomy on Intraocular Pressure in Pigment Dispersion Syndrome. Ophthalmology.

[54] Ritch R., Liebmann J., Robin A., Pollack I.P., Harrison R., Levene R.Z., Hagadus J. (1993). Argon Laser Trabeculoplasty in Pigmentary Glaucoma. Ophthalmology.

[55] Robin A.L., Pollack I.P. (1983). Argon Laser Trabeculoplasty in Secondary Forms of Open-Angle Glaucoma. Arch. Ophthalmol..

[56] Lunde M.W. (1983). Argon Laser Trabeculoplasty in Pigmentary Dispersion Syndrome with Glaucoma. Am. J. Ophthalmol..

[57] Ayala M. (2014). Long-Term Outcomes of Selective Laser Trabeculoplasty (SLT) Treatment in Pigmentary Glaucoma Patients. J. Glaucoma.

[58] Hallaj S., Sinha S., Mehran N.A., Morrill A.M., Pro M.J., Dale E., Schmidt C., Kolomeyer N.N., Shukla A.G., Lee D. (2024). Intraocular Pressure Profile Following Selective Laser Trabeculoplasty in Pigmentary and Primary Open-Angle Glaucoma. Eur. J. Ophthalmol..

[59] Harasymowycz P.J., Papamatheakis D.G., Latina M., De Leon M., Lesk M.R., Damji K.F. (2005). Selective Laser Trabeculoplasty (SLT) Complicated by Intraocular Pressure Elevation in Eyes With Heavily Pigmented Trabecular Meshworks. Am. J. Ophthalmol..

[60] Scuderi G.L., Pasquale N. (2008). Laser Therapies for Glaucoma: New Frontiers. Progress in Brain Research.

[61] Torrado I.A., Martínez Córdoba C.J., Moreno Mazo S.E., Toquica J.E., Hernandez P. (2025). Intraocular Pressure Reduction in Patients Treated with Micropulse Laser Trabeculoplasty vs Selective Laser Trabeculoplasty. Indian J. Ophthalmol..

[62] Qing G.-P., Wang N.-L., Wang T., Chen H., Mou D.-P. (2016). Long-Term Efficacy of Trabeculectomy on Chinese Patients with Pigmentary Glaucoma: A Prospective Case Series Observational Study. Chin. Med. J..

[63] Kim E.-A., Law S.K., Coleman A.L., Nouri-Mahdavi K., Giaconi J.A., Yu F., Lee J.-W., Caprioli J. (2015). Long-Term Bleb-Related Infections After Trabeculectomy: Incidence, Risk Factors, and Influence of Bleb Revision. Am. J. Ophthalmol..

[64] (2007). CAT-152 Trabeculectomy Study Group. Factors Affecting the Outcome of Trabeculectomy. Ophthalmology.

[65] Akil H., Chopra V., Huang A., Loewen N., Noguchi J., Francis B.A. (2016). Clinical Results of Ab Interno Trabeculotomy Using the Trabectome in Patients with Pigmentary Glaucoma Compared to Primary Open Angle Glaucoma. Clin. Exper. Ophthalmol..

[66] Gassel C.J., Nasyrov E., Wenzel D.A., Voykov B. (2025). XEN45 Gel Stent in the Treatment of Pigmentary Glaucoma: A Two-Year Follow-Up. Eur. J. Ophthalmol..

[67] Łazicka-Gałecka M., Kamińska A., Gałecki T., Guszkowska M., Dziedziak J., Szaflik J., Szaflik J.P. (2022). Canaloplasty—Efficacy and Safety in an 18-Month Follow Up Period, and Analysis of Outcomes in Primary Open Angle Glaucoma Pigmentary Glaucoma and Pseudoexfoliative Glaucoma. Semin. Ophthalmol..

[68] Brusini P., Papa V. (2020). Canaloplasty in Pigmentary Glaucoma: Long-Term Outcomes and Proposal of a New Hypothesis on Its Intraocular Pressure Lowering Mechanism. JCM.

[69] Klamann M.K.J., Gonnermann J., Pahlitzsch M., Maier A.-K.B., Joussen A.M., Torun N., Bertelmann E. (2015). iStent Inject in Phakic Open Angle Glaucoma. Graefe’s Arch. Clin. Exp. Ophthalmol..

[70] Ferguson T.J., Ibach M., Schweitzer J., Karpuk K.L., Stephens J.D., Berdahl J.P. (2020). Trabecular Micro-bypass Stent Implantation with Cataract Extraction in Pigmentary Glaucoma. Clin. Exper. Ophthalmol..

[71] Jacobi P.C., Dietlein T.S., Krieglstein G.K. (2000). Effect of Trabecular Aspiration on Intraocular Pressure in Pigment Dispersion Syndrome and Pigmentary Glaucoma. Ophthalmology.

[72] Laroche D., Scheive M. (2022). How to Stop People from Going Blind from Glaucoma Using Early Cataract Surgery/Refractive Lensectomy and Microinvasive Glaucoma Surgery. Clin. Ophthalmol..

